# Development of a non-radiometric method for measuring the arterial input function of a ^11^C-labeled PET radiotracer

**DOI:** 10.1038/s41598-020-73646-4

**Published:** 2020-10-15

**Authors:** H. Umesha Shetty, Sami S. Zoghbi, Cheryl L. Morse, Aneta Kowalski, Jussi Hirvonen, Robert B. Innis, Victor W. Pike

**Affiliations:** 1 Molecular Imaging Branch, National Institute of Mental Health, National Institutes of Health (NIMH/NIH), Building 10, Room B3C351, 10 Center Drive, MSC 1003, Bethesda, MD 20892 USA; 2 Department of Radiology and Turku PET Centre, University of Turku and Turku Central Hospital, 20520 Turku, Finland

**Keywords:** Biological techniques, Drug discovery, Neuroscience, Medical research, Chemistry

## Abstract

Positron emission tomography (PET) uses radiotracers to quantify important biochemical parameters in human subjects. A radiotracer arterial input function (AIF) is often essential for converting brain PET data into robust output measures. For radiotracers labeled with carbon-11 (*t*_1/2_ = 20.4 min), AIF is routinely determined with radio-HPLC of blood sampled frequently during the PET experiment. There has been no alternative to this logistically demanding method, neither for regular use nor validation. A ^11^C-labeled tracer is always accompanied by a large excess of non-radioactive tracer known as carrier. In principle, AIF might be obtained by measuring the molar activity (*A*_m_; ratio of radioactivity to total mass; Bq/mol) of a radiotracer dose and the time-course of carrier concentration in plasma after radiotracer injection. Here, we implement this principle in a new method for determining AIF, as shown by using [^11^C]PBR28 as a representative tracer. The method uses liquid chromatography-tandem mass spectrometry for measuring radiotracer *A*_m_ and then the carrier in plasma sampled regularly over the course of a PET experiment. *A*_m_ and AIF were determined radiometrically for comparison. The new non-radiometric method is not constrained by the short half-life of carbon-11 and is an attractive alternative to conventional AIF measurement.

## Introduction

Positron emission tomography (PET) is a uniquely valuable molecular imaging modality for noninvasively exploring physiology and biochemistry in health and disease^1,2^, and has an expanding role in drug development^3^ and medical diagnosis^4^. PET has notable importance for neuropsychiatric research both for the study of pathophysiology and for drug development. Appropriately designed radiotracers^5^ permit sensitive imaging and quantification of many of the proteins in brain^6^ that are implicated in neuropsychiatric^7^, neurological^8^, and neurodegenerative disorders^9^, as well as substance dependence^10^. These proteins include various neurotransmitter receptors, transporters, enzymes, and amyloid plaques^3^. PET radiotracers may also be used to verify protein target engagement by experimental drugs and how target engagement varies with dosing regimen^11^. Such information can be critical for establishing meaningful clinical trials. Short-lived carbon-11 (*t*_1/2_ = 20.4 min) or fluorine-18 (*t*_1/2_ = 110 min) are the two most commonly used radionuclides for labeling radiotracers for PET imaging of brain^5^.

Typically, in each PET scanning session, measurement of the radiometabolite-corrected arterial input function (AIF) of the radiotracer is required for use in conjunction with a biomathematical model to robustly quantify a radiotracer target within brain^12^. Nearly all PET radiotracers generate radiometabolites in plasma^5^. The AIF is the time-course of non-metabolized radiotracer in plasma. Virtually all AIF measurement has been based on a single methodology, namely fast radio-high performance liquid chromatography (radio-HPLC) separation of the parent radiotracer from radiometabolites in plasma from multiple blood samples taken serially throughout a PET scanning session for radiometric quantification. When imaging with a ^11^C-labeled PET tracer, the time available for measuring AIF by this means is severely constrained to a few half-lives (typically ~ 90 min). This is very logistically demanding. Whereas the dose of a radiotracer administered to a human subject (~ 750 MBq) is measured with an ionization chamber, the low levels of radioactivity found in plasma samples (of the order of kBq) are typically measured with a sensitive γ-counter. The accuracy of radioactivity measured with an ionization chamber or γ-counter depends, among other factors, on the surrogate radioisotope(s) used to calibrate the instruments^13,14^. Notably, no alternative method has ever been available to validate AIFs measured with the conventional radiometric technique. A method for AIF measurement that avoids the severe time constraint imposed by fast decaying radioactivity would be invaluable.

A ^11^C-labeled PET radiotracer is always accompanied by a matching non-radioactive tracer known as carrier. Although the carrier mass is typically small (< 5 nmol) in any administered radiotracer dose, it is always much larger than the mass of the radiotracer, usually by about three orders of magnitude. In principle, AIF may be obtained by measuring the molar activity (*A*_m_; ratio of radioactivity to combined mass of tracer and carrier; Bq/mol) of a radiotracer dose and the time-course of the very low concentrations of carrier in plasma after radiotracer injection.

**Figure 1:**
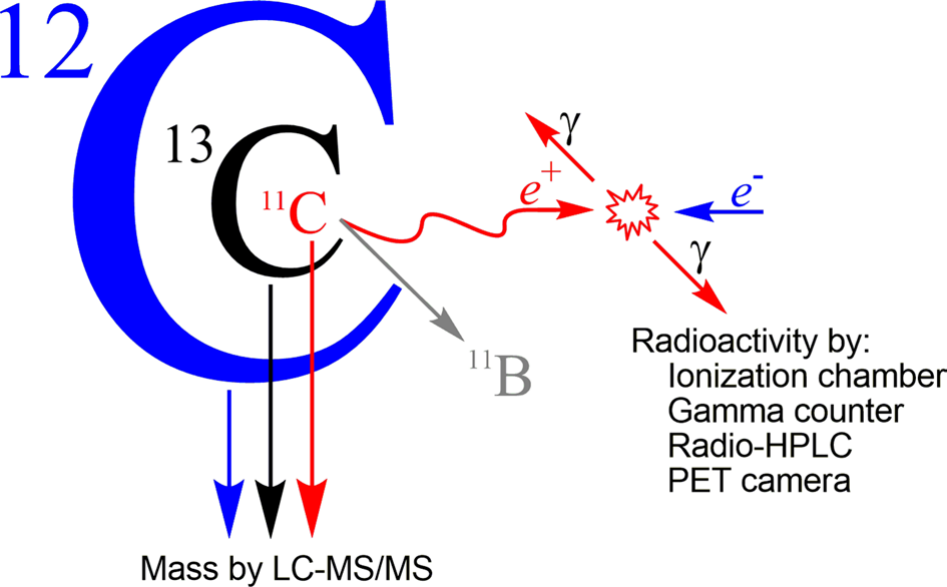
Relative abundances of carrier ([^12^C]_i_ + [^13^C]_i_) and carbon-11 ([^11^C]_i_) species in a ^11^C-labeled tracer: [^12^C]_i_ 98.9%; [^13^C]_i_ ~ 1.1%; and [^11^C]_i_ typically < 0.1%. In this report, LC–MS/MS was used to measure these three species in order to derive the relationship between tracer mass (through activity derived from Avogadro’s number and decay constant) and radioactivity.

An earlier study from our laboratory demonstrated that liquid chromatography-tandem mass spectrometry (LC–MS/MS) has sufficient sensitivity to detect not only the non-radioactive isotopologues (i.e., ^12^C and ^13^C species) in a ^11^C-labeled tracer but also the relatively low mass radioactive isotopologue (i.e., the ^11^C species)^15^. Here, in this study, we further explored the use of sensitive LC–MS/MS to measure each of the three carbon isotopologues in a PET radiotracer dose, which we here denote [^11^C]_i_, [^12^C]_i_, and [^13^C]_i_, (Fig. 1), and the application of such measurements to human plasma samples for AIF determination. We furthermore compared the resultant *A*_m_ and AIF data with those determined by the conventional radiometric method for the same ^11^C-labeled radiotracer, using [^11^C]PR28 as the principal example.

To develop an LC–MS/MS method for AIF determination, we needed to show that LC–MS/MS has (1) sufficient sensitivity to accurately measure the ratio of all three isotopologues in a radiotracer dose, (2) adequate sensitivity and specificity to measure the low level of carrier from the radiotracer in human plasma after intravenous administration of the radiotracer at a useful molar activity, especially over the whole time course of PET imaging (typically 90 min), and (3) that the obtained AIF is reliable and accurate. We performed detailed experiments to establish these aims.

## Results

### LC–MS/MS investigation of the carrier in ^11^C-labeled tracers: ^13^C to ^12^C ratio at the radiolabeling site and dependence of this ratio on *A*_m_

As part of method development, we set out to measure the ratio of ^13^C to ^12^C in the carrier of some ^11^C-labeled tracers to determine whether there was any variability that might impact on the proposed use of LC–MS/MS for measuring *A*_m_ and AIF. We showed that we could measure simultaneously all three isotopologues, [^11^C]_i_, [^12^C]_i_, and [^13^C]_i_, in radiotracers with high *A*_m_ values and, two isotopologues, [^11^C]_i_ and [^13^C]_i_, when *A*_m_ is in low to moderate range. The ratio of ^13^C to ^12^C in the natural environment is about 1.1% but shows small variations that depend on carbon source^16^. For the determination of *A*_m_ values, we proposed to measure two isotopologues only, [^11^C]_i_, and [^13^C]_i_. This approach avoids detector saturation with the much more abundant [^12^C]_i,_ especially for samples with *A*_m_ at the lower end of the normal range. To calculate the amount of [^12^C]_i_ from a measurement of [^13^C]_i_, their exact ratio in a sample of the compound would need to be known. This ratio was first measured in natural abundance reference compounds for four of the studied PET radiotracers (specifically, three TSPO radiotracers—[^11^C]PBR28^17^, [^11^C](*R*)-PK11195^18^, and [^11^C]DPA713^19^—and one cyclic adenosine monophosphate (cAMP) phosphodiesterase-4 (PDE-4) radiotracer, [^11^C](*R*)-rolipram^20^). The obtained values were within the expected range of about 1.1% per carbon atom in a measured product ion (reference standards, Table 1).

**Table 1:**
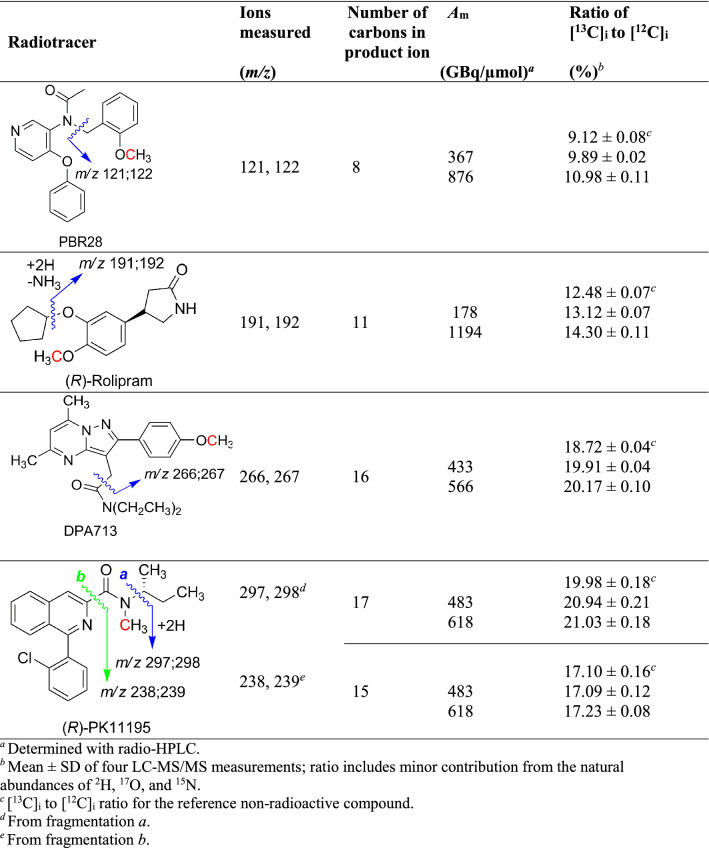
Ratios of [^13^C]_i_ to [^12^C]_i_ in the carrier of four ^11^C-labeled PET radiotracers produced at two different *A*_m_ values, and in the respective reference compounds. The red carbon denotes the site that is labeled with carbon-11 in each radiotracer.

^a^Determined with radio-HPLC.

^b^Mean ± SD of four LC–MS/MS measurements; ratio includes minor contribution from the natural abundances of ^2^H, ^17^O, and ^15^N.

^c^[^13^C]_i_ to [^12^C]_i_ ratio for the reference non-radioactive compound.

^d^From fragmentation a*.*

^e^From fragmentation b*.*

However, when this method was applied to the carrier present in prepared radiotracer doses, we observed [^13^C]_i_ to [^12^C]_i_ ratios that were well above the normal range for natural abundance (Table 1). These findings showed that ^13^C enrichment had occurred during radiotracer production, which we now attribute to a known nuclear reaction, ^14^N(p,2n)^13^C, that would co-exist with the ^14^N(p,α)^11^C reaction during the cyclotron production of carbon-11 through irradiation of nitrogen with 16 MeV protons (see “Discussion” section)^21^. In our production setting, the *A*_m_ value of a PET radiotracer strongly reflects the amount of radioactivity produced during cyclotron irradiation, which in turn depends on the integrated proton beam current (μA × min)^22^. Consequently, we predicted that the [^13^C]_i_ to [^12^C]_i_ ratio would increase with the measured *A*_m_ value of the ^11^C-labeled tracer. In each of four radiotracers, when measuring the fragment ion that contained the radiolabeling site in two separately prepared doses, the [^13^C]_i_ to [^12^C]_i_ ratio was higher in the dose that had the higher *A*_m_ value (determined radiometrically) (Table 1). One radiotracer (*R*)-PK11195 gave abundant product ions (*m/z* 238; 239) that lacked the ^11^C-labeling site (i.e., lacked the amido *N*-methyl group). The [^13^C]_i_ to [^12^C]_i_ ratio for these ions was found to be in the range of natural abundance and invariant with the *A*_m_ value of the radiotracer preparation (Table 1), thereby affirming that changes in ^13^C-enrichment were confined to the fragment containing the ^11^C-labeling site.

The data from Table 1 were used to plot the increase in the ratio of [^13^C]_i_ to [^12^C]_i_ for the carrier in all eight radiotracer productions versus *A*_m_ value. These values and the corresponding *A*_m_ values were found to be strongly correlated (*r* = 0.895; *p* < 0.003) (Supplementary Fig. S1).

### Determination of radiotracer *A*_m_ by LC–MS/MS alone

*A*_m_ values for [^11^C]PBR28 preparations were measured with LC–MS/MS by isolating and monitoring the isotopologue pair, [^11^C]_i_ and [^13^C]_i_, as previously described for ^11^C-labeled tracers^15^. In that study, the ratio of [^13^C]_i_ to [^12^C]_i_ was taken to be the fixed value measured in reference PBR28. Here, in a refinement of this procedure, the ratio of [^13^C]_i_ to [^12^C]_i_ in the carrier of each [^11^C]PBR28 preparation was used to calculate the ^12^C-peak area from which *A*_m_ values could then be derived. In the case of a few [^11^C]PBR28 preparations, where the *A*_m_ values were relatively high (905–1124 GBq/µmol), LC–MS/MS successfully isolated and monitored all three carbon isotopologues simultaneously ([^11^C]_i_, [^12^C]_i_, and [^13^C]_i_) (Supplementary Fig. S2). This direct measurement of all three types of isotopologue yielded *A*_m_ values that closely matched those obtained by measuring only the two isotopologues [^11^C]_i_ and [^13^C]_i_. Therefore, measurement of [^11^C]_i_ and [^13^C]_i_ alone plus a separate measurement of the ^13^C to ^12^C ratio in a radiotracer dose sufficed to provide an accurate *A*_m_ value.

### Plot of [^13^C]_i_ to [^12^C]_i_ ratio versus *A*_m_ (by LC–MS/MS) for [^11^C]PBR28

**Figure 2:**
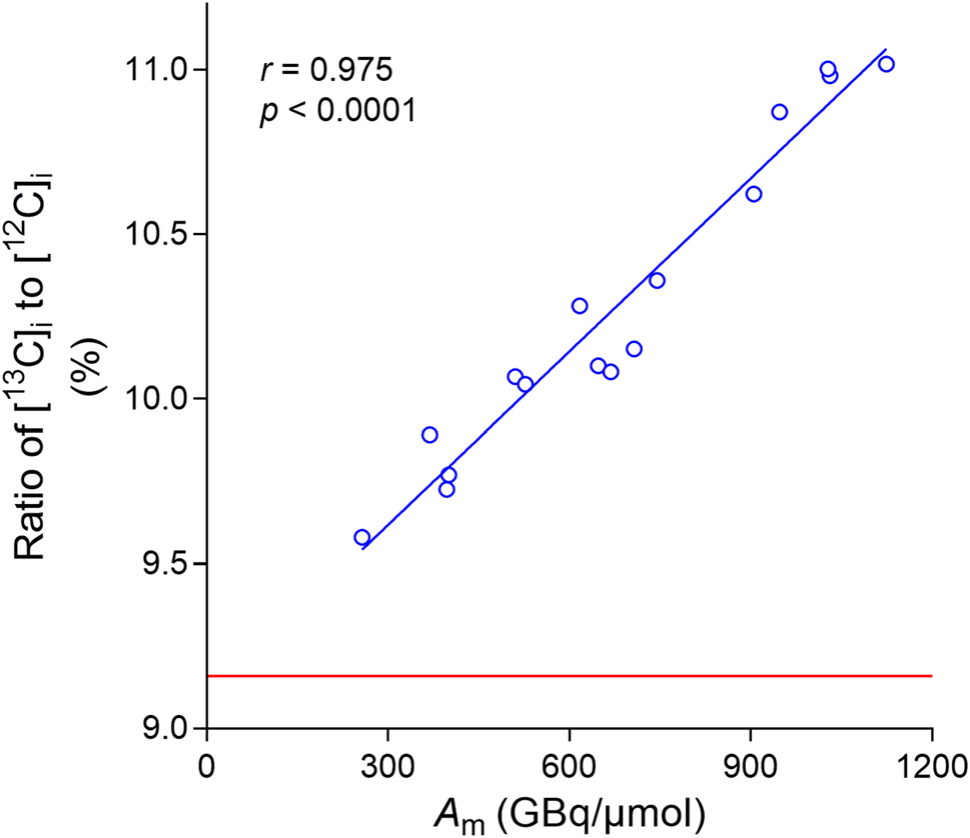
The percentage ratio of [^13^C]_i_ to [^12^C]_i_ in carrier increased linearly with *A*_m_ value (determined by LC–MS/MS) for [^11^C]PBR28 preparations. The red line represents the mean value of the ratio in reference PBR28.

*A*_m_ values, including the ratio of [^13^C]_i_ to [^12^C]_i_, were determined in 16 preparations of [^11^C]PBR28. Variations in radiosynthesis time were negligible (36.3 ± 0.82 min; mean ± SD; *n* = 16) and dose radioactivity was therefore decay-corrected to the end of each synthesis. As a control, the ratio of [^13^C]_i_ to [^12^C]_i_ was also measured in reference (natural abundance) PBR28 on each occasion of radiotracer analysis. The ratios of [^13^C]_i_ to [^12^C]_i_ for [^11^C]PBR28 preparations correlated strongly with *A*_*m*_ values determined with LC–MS/MS (*r* = 0.975; *p* < 0.0001; *n* = 16) (Fig. 2). The *Y*-axis intercept of 9.09% (*A*_m_ = 0) for this curve was almost identical to the mean ratio of [^13^C]_i_ to [^12^C]_i_ measured for reference PBR28 (9.16 ± 0.05%; mean ± SD; *n* = 16; represented by the red line in Fig. 2). The small standard deviations in the latter value and those in Table 1 demonstrate the high precision with which such ratios could be determined with LC–MS/MS.

### Comparison of LC–MS/MS and radiometric methods for measuring *A*_m_

The *A*_m_ values of each of 15 preparations of [^11^C]PBR28 were measured in three different ways: with LC–MS/MS alone (Method 1) ; with carrier measured with LC–MS/MS and radioactivity measured in a calibrated γ-counter (Method 2), and with the conventional radiometric method based on radioactivity dose measured in an ionization chamber and carrier measured with HPLC (Method 3) (Supplementary Methods). A scatter plot of the percentage difference between the *A*_m_ values measured with LC–MS/MS alone and each of the two radiometric methods is displayed in Supplementary Fig. S3. When compared with LC–MS/MS (Method 1), measurement with an ionization chamber (Method 2) gave 1.5 ± 10.1% lower *A*_m_ values, and measurement with a γ-counter (Method 3) gave 33.2 ± 3.3% higher *A*_m_ values.

### Comparison of radioactivity measurements made with LC–MS/MS with those of γ-counter

Radioactivity in a sample of [^11^C]PBR28 was measured with a calibrated γ-counter*.* The carrier [^12^C]_i_ in the same sample was then quantified with LC–MS/MS using an internal standard of [^13^C,^2^H_3_]PBR28 to calibrate the LC–MS/MS response. Radioactivity based on the mass of [^11^C]_i_ was calculated using the concentration of the carrier and the *A*_m_ value of [^11^C]PBR28 determined with LC–MS/MS. Measurement with a γ-counter gave 31.8 ± 3.8% (*n* = 24) higher radioactivity values than LC–MS/MS. Data from the analysis of six [^11^C]PBR28 preparations are shown in Table 2. The substantial difference between the radioactivity obtained by direct counting and through the mass of [^11^C]_i_ was independent of the *A*_m_ value (200–1028 GBq/µmol) and also independent of whether two istopologues ([^11^C]_i_ and [^13^C]_i_) or three isotopologues ([^11^C]_i_, [^12^C]_i_ and [^13^C]_i_) were analyzed. Thus, differences in radioactivity estimates from γ-counting and LC–MS/MS accounted for all the *A*_m_ discrepancies shown in Supplementary Fig. S3b.

**Table 2:** Comparison of [^11^C]PBR28 radioactivity data from γ-counter and from LC–MS/MS.

[^11^C]PBR28 preparation	Carrier by LC–MS/MS (pmol)^a^	*A*_m_ by LC–MS/MS (GBq/µmol)^b^	Radioactivity by		Difference in radioactivity (%)^d^
			LC–MS/MS (kBq)^c^	γ-counter (kBq)	
1	15.2	200.4	3040	4019	32.2
2	5.56	631.1	3510	4817	37.2
3	12.1	383.5	4645	6310	35.8
4	1.75	904.9	1580	2092	32.4
5	2.90	1124	3261	4249	30.3
6	2.95	1028	3036	4150	36.7

^a^Measured using [^13^C,^2^H_3_]PBR28 as an internal standard.

^b^*A*_m_ value by LC–MS/MS of [^11^C]_i_ and [^13^C]_i_ (1–3), and of the triad [^11^C]_i_, [^12^C]_i,_ and [^13^C]_i_ (4–6).

^c^Radioactivity calculated from the *A*_m_ and carrier, each measured with LC–MS/MS.

^d^From that determined with LC–MS/MS.

### LC–MS/MS of carrier in human plasma

**Figure 3:**
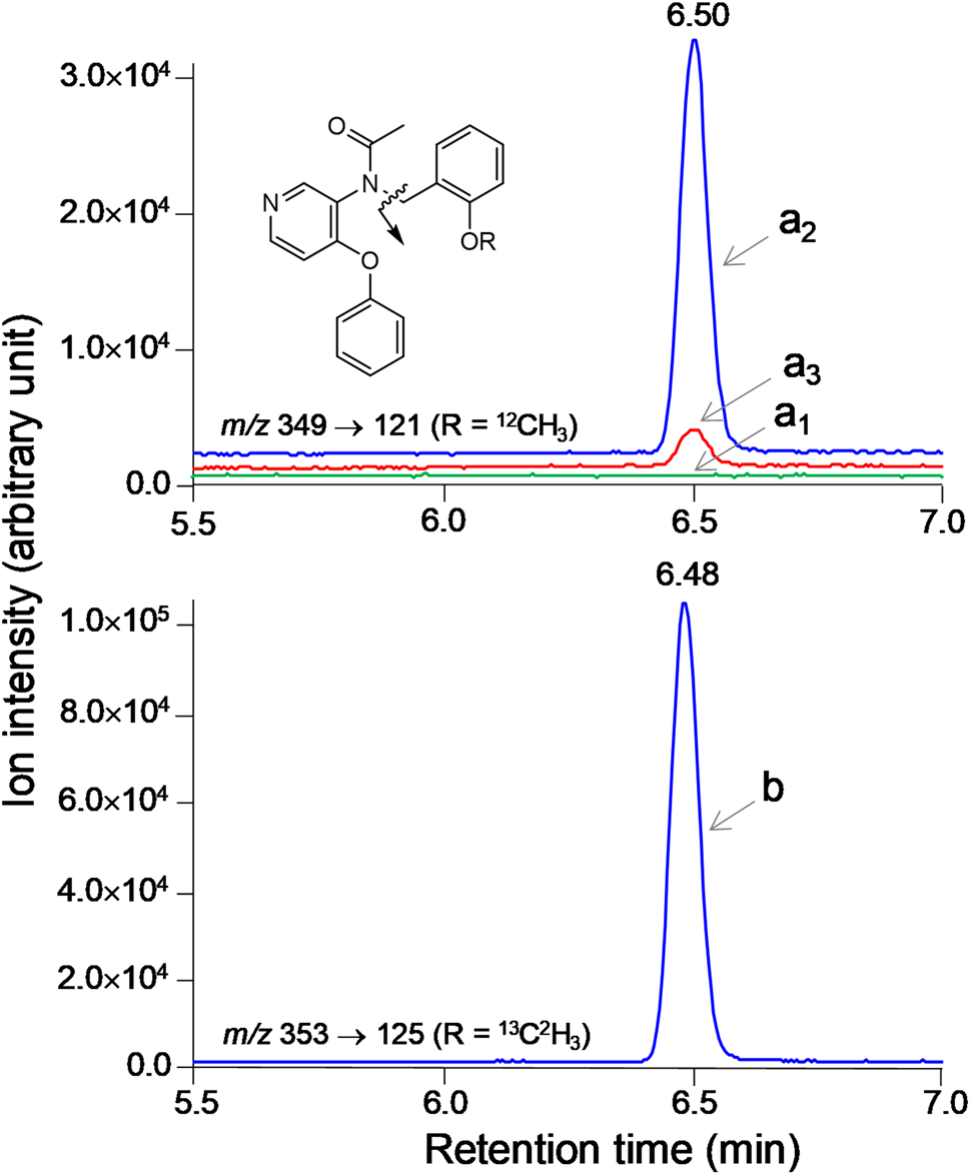
LC–MS/MS ion chromatograms (*m/z* = 121; [^12^C]_i_) for carrier PBR28 in arterial plasma sampled from a human subject injected intravenously with [^11^C]PBR28 for PET imaging. a_1_: baseline. Peaks a_2_ and a_3_: carrier PBR28 peaks at 10 min (71.8 pM) and 90 min (6.34 pM) after injection, respectively. Peak b in ion chromatogram (*m/z* = 125) is from the analogous transition in the internal standard, [^13^C,^2^H_3_]PBR28 (284 pM).

By using [^13^C,^2^H_3_]PBR28 as an internal standard, a LC–MS/MS method was developed to quantify the very low amounts of PBR28 carrier in the plasma of eight human subjects who had been injected intravenously with [^11^C]PBR28 for PET scanning. Figure 3 shows ion chromatograms from the analysis of plasma at baseline, and at 10 and 90 min after intravenous injection of [^11^C]PBR28 (710 MBq; *A*_m_: 383.5 GBq/µmol) in one subject. The plasma matrix did not interfere with the ionization or detection of carrier PBR28 or of the internal standard, and no interfering peak was observed in the ion chromatograms. The measured ratios of carrier to internal standard peak area gave the true concentrations of carrier PBR28 from the linear calibration curve (Supplementary Fig. S4).

### LC–MS/MS measurement for AIF determination

The *A*_m_ values of [^11^C]PBR28 doses administered to three different human subjects for PET imaging were measured with LC–MS/MS. These values were used to convert arterial plasma [^11^C]PBR28 radioactivity from conventional AIF measurements at four timepoints into picomolar concentrations of carrier PBR28. This allowed the two datasets (radiometric and LC–MS/MS) to be compared. The plasma concentration of carrier PBR28 decreased as the *A*_m_ value of the injected radiotracer increased, as would be expected for similar administered amounts of radioactivity per weight of subject. Plasma concentrations of carrier PBR28 calculated from radiometric measurement of [^11^C]PBR28 radioactivity were higher than those measured with LC–MS/MS. After correcting for the systematic difference between measurements, the carrier PBR28 concentrations determined by radiometric and LC–MS/MS methods were highly comparable (Table 3), including those at very low levels of carrier concentration (~ 5 pM). Further experiments showed that the LC–MS/MS method had adequate sensitivity to measure [^11^C]PBR28 in plasma at 23 timepoints for up to 90 min following intravenous injection of the radiotracer at different molar activities.

**Table 3:** Carrier PBR28 concentration (pM) in arterial plasma after [^11^C]PBR28 injection.

*A*_m_ (GBq/µmol)^a^	Time after injection (min)	Carrier concentration (pM)		
		Radiometric^b^		LC–MS/MS
		A	B	
138.6	1.25	2349	1726	1790
	2.50	416.8	306.2	303.9
	20.00	52.20	38.36	36.02
	59.98	29.81	21.90	21.83
200.4	1.23	1551	1173	1097
	2.48	304.6	230.4	214.6
	20.18	50.84	38.46	32.31
	60.00	17.41	13.17	11.76
385.9	1.25	773.8	599.9	594.1
	2.50	140.1	108.6	103.7
	20.00	16.48	12.77	11.10
	60.00	7.288	5.649	5.158

^a^Determined with LC–MS/MS for each radiometric and LC–MS/MS method.

^b^From radiometric measurement before (A) and after (B) the correction of radioactivity (for the systematic difference between LC–MS/MS and γ-counter measures).

### Comparisons of AIFs for [^11^C]PBR28 from LC–MS/MS and radiometric methods

**Figure 4:**
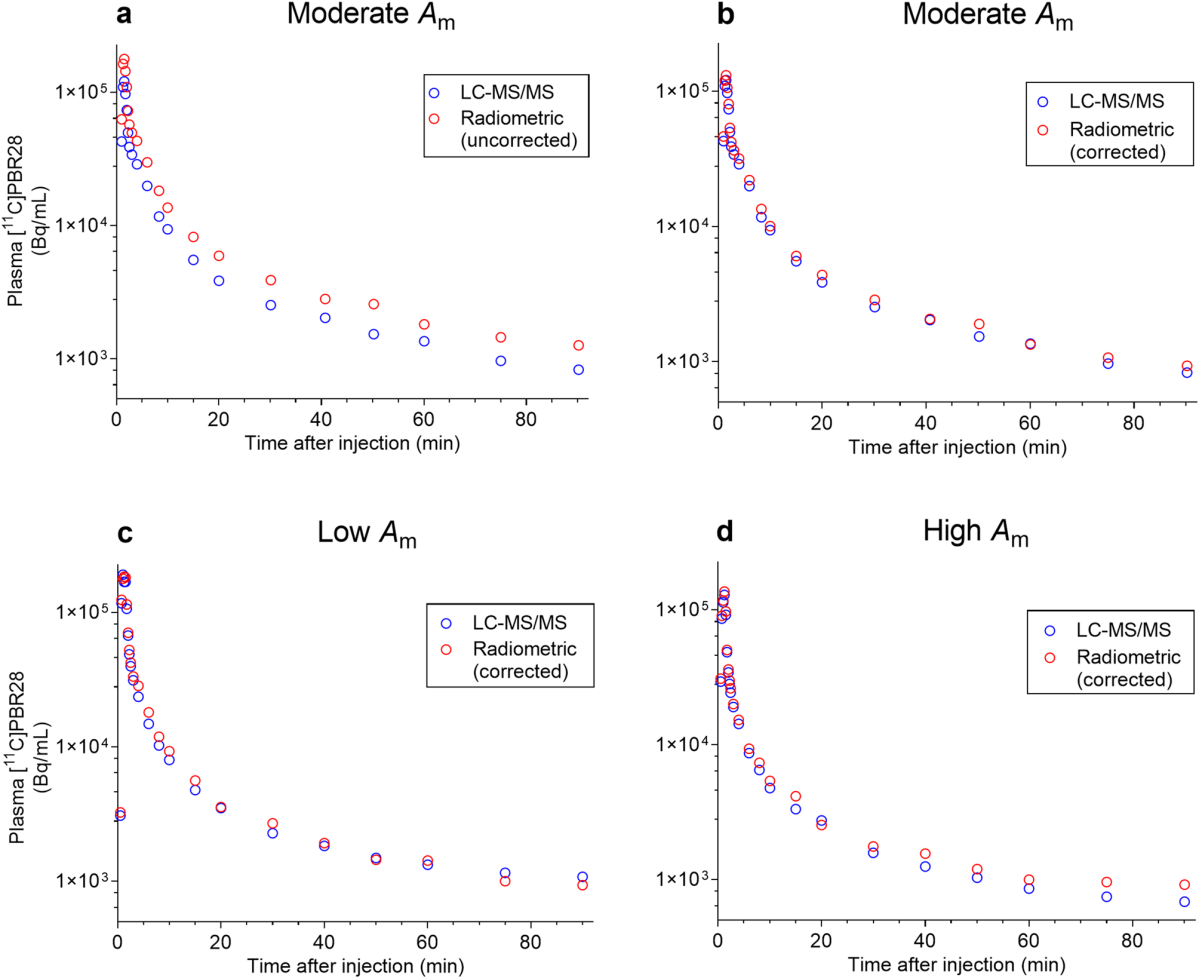
Examples of AIFs determined in human subjects with radiometric and LC–MS/MS methods for [^11^C]PBR28. (**a**) AIFs for one subject injected with [^11^C]PBR28 (10.1 MBq/kg; injected mass 92 pmol/kg, i.v.) with an *A*_m_ of 383.5 GBq/µmol. The AIF from radiometric method is without correction of γ-counter measured radioactivity for the systematic difference with LC–MS/MS. (**b**) Comparisons of AIFs from the same experiment after correction of γ-counter measured radioactivity. (**c**) AIFs from radiometric (corrected) and LC–MS/MS measurements in another human subject injected with [^11^C]PBR28 (10.7 MBq/kg; injected mass 229 pmol/kg, i.v.) at a lower *A*_m_ of 141.8 GBq/µmol. (**d**) AIFs from the same methods in a subject injected with radiotracer (9.5 MBq/kg; injected mass 44 pmol/kg, i.v.) at a higher *A*_m_ of 631.1 GBq/µmol. Note all data are for unchanged radiotracer alone (i.e., radiometabolites are excluded).

The carrier PBR28 concentrations in plasma samples measured with LC–MS/MS were transformed into radioactivity data using *A*_m_ measured with LC–MS/MS. An example of a log-linear plot of plasma [^11^C]PBR28 radioactivity versus time (i.e., AIF) from the new LC–MS/MS method and the conventional radiometric method for a human subject injected with [^11^C]PBR28 at a moderately high *A*_m_ value (i.e., with low carrier) is illustrated in Fig. 4a. In this example, and in seven other PET experiments with [^11^C]PBR28, the log-linear plots of AIFs from LC–MS/MS ran nearly parallel below the AIFs obtained via the conventional radiometric method. After correcting the plasma radioactivity measured with the γ-counter for the systematic difference between the methods (as described above, see Supplementary Fig. S3), the AIF measured with LC–MS/MS became virtually superimposed with that measured radiometrically (Fig. 4b) for each example. AIF plots from radiometric and LC–MS/MS measurements for human subjects administered with [^11^C]PBR28 of a low *A*_m_ of 141.8 GBq/µmol and a high *A*_m_ of 631.1 GBq/µmol) are shown in Fig. 4c,d, respectively.

To examine the impact of different types of AIF measurement on the input function for kinetic modeling of PET data, we compared areas under the curve (AUCs) for the plasma time-activity curves. On average, AUC calculated from LC–MS/MS was 31% lower than that calculated with the radiometric method (*t* = − 11.9, *p* < 0.001), and 8% lower than that calculated with the corrected radiometric method (*t* = − 6.0, *p* < 0.001) (Table 4). VAR, Pearson correlation coefficient, and ICC, were respectively 37%, 0.97, and 0.07 for LC–MS/MS versus direct radiometric method, and 8%, 0.99, and 0.91 for LC–MS/MS versus the corrected radiometric method.

**Table 4:** AUCs (kBq × min/mL) for the plasma time-activity curves from LC–MS/MS and radiometric measurements.

Human subject	AUCs (kBq × min/mL)			Difference (%)^b^	
	LC–MS/MS	Radiometric^a^			
		A	B	A	B
1	328	470	356	36	8
2	308	485	351	44	13
3	329	437	339	28	3
4	332	465	342	33	3
5	334	500	365	40	9
6	460	686	505	39	9
7	336	517	380	42	12
8	518	730	559	34	8
Mean ± SD	368 ± 77	536 ± 109	399 ± 84	37 ± 5.3	8.1 ± 3.6
RSD (%)	21	20	21		

^a^AUCs from radiometric measurements before (A) and after (B) the correction of radioactivity (for the systematic difference between LC–MS/MS and γ-counter measures).

^b^Between the AUC from LC–MS/MS and that from the radiometric method for AUC dataset A and AUC dataset B.

## Discussion

This study assessed the feasibility of using LC–MS/MS for measuring AIF in human subjects undergoing PET scanning with a ^11^C-labeled radiotracer. LC–MS/MS analysis was found to provide a convenient and sensitive method for measuring the AIF of a ^11^C-labeled tracer without measuring its radioactivity. This method can be performed on multiple blood samples without the time and logistical constraints of the conventional radiometric method. The *A*_m_ measured during the production of a radiotracer may be used to transform plasma carrier concentrations into AIF radioactivity data.

During the LC–MS/MS analysis of the triad, [^11^C]_i_, [^12^C]_i_, and [^13^C]_i_ in all four tested radiotracers, we observed a higher [^13^C]_i_ to [^12^C]_i_ ratio than in the respective non-radioactive standard (Table 1). The MS/MS technique demonstrated that ^13^C-enrichment had occurred in the carrier at the same position that had been labeled with carbon-11 during radiotracer synthesis. As clearly shown for [^11^C]PBR28, the degree of isotopic enrichment correlated with *A*_m_ value. Therefore, the augmented [^13^C]_i_ to [^12^C]_i_ ratio was not due to an isotope effect in the synthesis or purification of the radiotracer. The carbon-11 for labeling each radiotracer was produced by the ^14^N(p,α)^11^C reaction on nitrogen with a 16 MeV beam of protons which degrade in energy on progressing through the gas target. An explanation for the *A*_m_-related ^13^C-enrichment is the co-occurrence of the ^14^N(p,2p)^13^C reaction. For irradiations of nitrogen gas with 13.2 MeV protons, the ^14^N(p,2p)^13^C reaction has a total cross section of 74.2 mb (millibarn) which is very similar to that for the ^14^N(p,α)^11^C reaction (68.9 mb)^23^. Therefore, the mass of generated carbon-13 is expected to be similar to the mass of carbon-11 produced. For a typical 40-min irradiation producing about 75 GBq of carbon-11, this amount would be about 10 nmol, or roughly enough to explain the ^13^C-enrichment seen for carrier in doses of [^11^C]PBR28.

The *A*_m_ value of a radiotracer needs to be determined accurately in order to derive mass of carrier from radioactivity or vice versa. A previous study from our laboratory reported an MS/MS technique for isolating [^11^C]_i_ and [^13^C]_i_ to measure the *A*_m_ values for ^11^C-labeled tracers^15^. Each *A*_m_ value was calculated using a constant [^13^C]_i_ to [^12^C]_i_ ratio that had been measured for the reference natural abundance ligand. In the present study, the *A*_m_ value for [^11^C]PBR28 was measured according to the same principle except that the specific [^13^C]_i_ to [^12^C]_i_ ratio of the carrier in the radiotracer was used for greater accuracy.

The sensitivity and dynamic range of MS/MS was found to be adequate to measure all three isotopologues in [^11^C]PBR28 with relatively high *A*_m_ values (~ 1000 GBq/µmol) (Supplementary Fig. S2). Such radiotracer preparations allowed injection of more diluted sample into the LC–MS/MS and thus measurement of carrier [^12^C]_i_ without saturating the detector. The *A*_m_ values determined from [^11^C]_i_ and [^13^C]_i_, as well as the [^13^C]_i_ to [^12^C]_i_ ratio, were found to be valid because direct measurement of the [^11^C]_i_, [^12^C]_i_, and [^13^C]_i_ triad yielded similar results (Table 2). The *A*_m_ value measured with a γ-counter was used to compare these two sets of data.

These findings further demonstrated that the LC–MS/MS technique can isolate and measure very low kBq levels of [^11^C]_i_ and, consequently, that this capability can be used to evaluate the accuracy of radioactivity measurements from radiation detectors such as ionization chambers and γ-counters. Building on this work, we compared the *A*_m_ values of [^11^C]PBR28 measured with LC–MS/MS with those determined using an ionization chamber and a γ-counter. The *A*_m_ (mean ± SD) value measured by ionization chamber was close to that obtained via LC–MS/MS, whereas that obtained via γ-counter was appreciably higher. Thus, comparing the three sets of *A*_m_ data revealed a significant difference in radioactivity estimates between the two commonly used radiometric methods.

Typically, an ionization chamber is calibrated for measuring carbon-11 with a pair of surrogate radioisotope standards, ^137^Cs (*t*_1/2_ = 30.17 years; β^−^, γ 662 keV) and ^57^Co (*t*_1/2_ = 271.79 days; ε, γ 122,136 keV), and a γ-counter with a different standard, ^68^Ge (*t*_1/2_ = 270.8  days; decays to ^68^Ga; *t*_1/2_ = 67.6 min, β^+^, ε, γ). Clearly, these surrogate isotopes have decay modes that are very different from those of ^11^C (β^+^, ~ 99.8%). Moreover, the accuracy of radioactivity measured with an ionization chamber or γ-counter is well known to be influenced by sample volume and geometry effects^14,24,25^. Studies seeking to measure positron-emitters in ionization chambers have been conducted with fluorine-18 (*t*_1/2_ = 109.8 min)^24,25^, but none have used shorter-lived carbon-11. Indeed, we have previously observed in our facility that identical ionization detectors calibrated in the same way with the same surrogate standards can give estimates of carbon-11 radioactivity differing by up to 12%^15^. During the course of the present study, we thoroughly investigated whether such differences could be ascribed to detector dead-time and linearity, sample geometry, and volume effects, to the material of the sample container (glass or polypropylene), or to measurement time. None of these factors accounted for the observed differences. These results underscore the role that a sensitive MS/MS technique may play in checking radioactivity measured with radiometric techniques. Notably, the MS/MS technique obviates sample volume and geometry concerns.

In the conventional radiometric measurement of AIF, plasma radioactivity is measured with a γ-counter and then corrected for the contribution of radiometabolites, as determined with radio-HPLC analysis^17,18^ (for radiochromatogram, Supplementary Fig. S5). The accuracy of AIF determined in this manner depends on the calibration of the γ-counter. The AIF data so generated can be compared with those from the LC–MS/MS of the carrier if equivalence between measured radioactivity and mass of [^11^C]_i_ has been demonstrated. Here, radioactivity based on the mass of [^11^C]_i_ was determined from the *A*_m_ and concentration of carrier (from LC–MS/MS) in a sample whose radioactivity had been measured with a γ-counter. The γ-counter measurement gave a higher value. Accordingly, it was necessary to adjust the radioactivity measured with a γ-counter to achieve a meaningful comparison of AIF determined from radiometric and LC–MS/MS methods.

LC–MS/MS, with the use of an internal standard labeled with stable isotopes (^13^C and deuterium), was able to quantify carrier PBR28 in small volumes of plasma (200 µL) taken from human subjects undergoing PET experiments with [^11^C]PBR28. Calibration of the method used reference PBR28 whose [^13^C]_i_ to [^12^C]_i_ ratio is lower than that of carrier PBR28. The unexpectedly skewed [^13^C]_i_ to [^12^C]_i_ ratio in the carrier was found to contribute negligible error (< 1%) to measurements of PBR28 concentrations. The LC–MS/MS method was sensitive and specific enough to measure carrier PBR28 up to 90 min after injection of radiotracer with *A*_m_ values in the range of 139 to 631 GBq/µmol. ^11^C-Labeled radiotracers are typically administered with molar activities at the lower end of this range. The entire range of quantification was achieved by injecting as little as 1/20th of each plasma sample onto the LC–MS/MS. If using a radiotracer with an exceptionally higher *A*_m_ value, quantification could still be achieved by increasing the injection volume, from for example 10 to 25 μL, or concentrating the plasma sample two-fold, although the LC procedure might consequently need some modification. In addition, it is expected that quantification limits would vary with the type of radiotracer carrier being measured.

With regards to measuring AIF for [^11^C]PBR28, when the radioactivity was corrected for the difference between radioactivity measured by γ-counter and by LC–MS/MS, the plasma concentration curves from the two methods matched (Fig. 4). The difference likely occurred because LC–MS/MS performs absolute quantification of the carrier whereas the γ-counter measures radioactivity relative to the surrogate radioisotope used for calibration. The radioactivity (Bq) is given by the product of the decay constant of the radionuclide and the number of un-decayed radioactive atoms. Thus, the LC–MS/MS measurement described here is expected to give absolute radioactivity, given that it is derived from the mass of [^11^C]_i_, the decay constant of carbon-11, and Avogadro’s number.

The AUCs for plasma time-activity curves from the LC–MS/MS method were 8.1 ± 3.6% (*n* = 8) lower than those from the radiometric method with corrected radioactivity. Nonetheless, the %RSD of AUCs calculated for 8 subjects was the same for the two methods and showed good correlation (Pearson *r* = 0.987; *p* = 0.01). Thus, the LC–MS/MS method is as reproducible as the radiometric method for measuring AIFs.

## Conclusion

The LC–MS/MS of fast-decaying PET radiotracers provides interchangeable mass and radioactivity data and offers the convenience of measuring AIF through the carrier of the radiotracer instead of radioactivity. The method, here exemplified with [^11^C]PBR28, circumvents possible radiometabolite interference and error due to volume and geometry effects associated with radiometric measurements. Potentially, this non-radiometric method might allow measurement of AIF on stored plasma samples by analytical service laboratories that perform LC–MS/MS quantifications. In such instances, AIFs in radioactivity unit can be derived from the *A*_m_ measured during production of the radiotracer. Taken together, the LC–MS/MS technique poses a convenient, non-radiometric, reproducible, and sensitive method for measuring AIF, deserving of widespread application in the expanding PET imaging field.

## Methods

The Supplementary Methods describe: (1) Materials; (2) Radiosynthesis; (3) Measurement of *A*_m_ using HPLC and an ionization chamber; (4) Technical aspects of AIF measurement by literature radiometric method; (5) Preparation of PBR28 and [^13^C,^2^H_3_]PBR28 internal standard (IS) stock solutions; (6) Extraction of carrier PBR28 in plasma for LC–MS/MS analysis; and (7) Recovery, matrix effect, stability, and reproducibility for LC–MS/MS quantification of carrier PBR28 in plasma.

### *A*_m_ of [^11^C]PBR28 by LC–MS/MS

An aliquot of a [^11^C]PBR28 preparation was diluted with LC mobile phase either 15-fold for measuring [^11^C]_i_ and [^13^C]_i_ or 40-fold for measuring [^11^C]_i_, [^12^C]_i_, and [^13^C]_i_. A sample (5 µL; *n* = 6 or 3) was injected onto LC–MS/MS (API 5000; Sciex; Redwood City, CA). Analysis was performed using LC method and MS/MS settings already described^15^. A second transition, *m/z* 349 → 121, was included in the method requiring acquisition of [^11^C]_i_, [^12^C]_i_, and [^13^C]_i_. In *A*_m_ measurements based on acquisition of [^11^C]_i_ and [^13^C]_i_, the ^13^C peak area of the carrier was converted into the ^12^C peak area using the ratio of [^13^C]_i_ to [^12^C]_i_ measured in the carrier, where [^13^C]_i_ includes [^12^C]_i_ having a single natural abundance ^2^H or ^17^O atom. *A*_m_ was determined from the peak areas of radioactive and carrier species as (A*)/(*A* + A*) × *A*_m_*, where A* is the sum of peak areas for [^11^C]_i_ and for the calculated area for the same species containing carbon-13, *A* is the sum of the peak areas for [^12^C]_i_ and [^13^C]_i_, and *A*_m_* is the theoretical carrier-free *A*_m_ of carbon-11 (3.413 × 10^20^ Bq/mol, the product of ln2/*t*_1/2_ and Avogadro’s number)^15^.

### Ratio of [^13^C]_i_ to [^12^C]_i_

The MS/MS instrument was tuned with reference ligands (PBR28, (*R*)-rolipram, DPA713, and (*R*)-PK11195). A method was set up to acquire [*M* + H]^+^ → product ion transitions for [^12^C]_i_ and [^13^C]_i_ (which includes ^12^C species containing a single ^2^H, ^15^N, or ^17^O atom of natural abundance) for each carrier, as follows: PBR28, *m/z* 349/350 → 121/122; (*R*)-rolipram, *m/z* 276/277 → 191/192; DPA713, *m/z* 367/368 → 266/267; and (*R*)-PK11195, *m/z* 353/354 → 297/298 (*a*) and 238/239 (*b*) (Table 1). Radiotracer samples were analyzed after full radioactive decay. Specifically, the sample was diluted (100–500 fold) and injected (5 µL; *n* = 4) onto the LC–MS/MS. Radiotracer’s carrier was chromatographed on a C18 column (2 × 20 mm, 3 µm; Phenomenex, Torrance, CA) using a similar water-acetonitrile (10 mM ammonium acetate or 0.2% acetic acid) gradient as previously reported^15^. The ratio of peak areas for product ion from [^13^C]_i_ to that from [^12^C]_i_, multiplied by 100, gave the ratio [^13^C]_i_ to [^12^C]_i_ as a % value.

Reference PBR28, (*R*)-rolipram, DPA713, and (*R*)-PK11195 were analyzed similarly, and the ratio of [^13^C]_i_ and [^12^C]_i_ was determined for each.

### Radioactivity and carrier in [^11^C]PBR28

In each of three glass vials, IS solution (2 ng) in dimethylformamide (DMF; 1 mL) was mixed with [^11^C]PBR28 preparation (3–5 µL) and the radioactivity counted with a calibrated γ-counter (model 1480 Wizard; Perkin-Elmer, Waltham, MA). After radioactivity decay, a 50 µL-aliquot of each sample was diluted with 450 µL of 1% acetic acid in aq. acetonitrile (50% v/v), and a sample was injected (5 µL) onto LC–MS/MS. Samples prepared by mixing the IS solution with known concentrations of reference PBR28 (10–0.3125 ng/mL DMF) were analyzed similarly to provide a calibration curve. Except for the LC column (3 μm; 2 × 50 mm; Phenomenex), the LC–MS/MS method used for the quantification of carrier PBR28 was the same as described for the plasma analysis (below).

### Arterial blood sampling from human subjects injected with [^11^C]PBR28

Blood samples used in this study were drawn from human subjects who were recruited by the Molecular Imaging Branch of the National Institute of Mental Health (NIMH). The study was approved by the National Institutes of Health (NIH) Combined Neurosciences Institutional Review Board (NCT 01547780; NCT 01851356; NCT 02233868). The selected participants signed informed consent before entering the study. After [^11^C]PBR28 injection, arterial blood samples were drawn at 15-s intervals up to 2 min and 30 s, and thereafter at 3, 4, 6, 8, 10, 15, 20, 30, 40, 50, 60, 75 and 90 min. Before injection of the radiotracer, baseline arterial blood was drawn, centrifuged, and plasma used for a control LC–MS/MS (carrier-free) measurement. All methods were performed in accordance with the relevant guidelines and regulations of the NIH.

### AIF for [^11^C]PBR28 by radiometric method

Arterial blood samples were immediately centrifuged after withdrawal and 1.5–2.0 min of transportation. Samples in Eppendorf tubes (cap sealed with Parafilm M) were centrifuged at 1800* g* for 2 min. The plasma was transferred to another tube and then split into two portions one for immediate radiometric AIF measurement, based on γ-counting of radioactivity and reverse phase HPLC analysis of plasma, as described previously^17,26^. Further technical details are provided in Supplementary Information. The second portion of plasma was mixed with IS and stored for separate determination of AIF by LC–MS/MS measurement of carrier PBR28 (see Supplementary Methods for sample preparation).

### AIF for [^11^C]PBR28 by LC–MS/MS quantification of carrier

The MS/MS was tuned as previously described^15^ and then set up to monitor *m/z* 349 [*M* + H]^+^ → 121 transition for carrier PBR28 and *m/z* 353 → 125 for the IS. A sample extracted from plasma (Supplementary Methods) was chromatographed at 40 °C on a C18 column (Kinetex, 2.6 μm; 3 × 75 mm; Phenomenex) eluted at 300 μL/min with a gradient of binary solvents (A:B) containing ammonium acetate (10 mM), where A was water/acetonitrile (90:10 v/v) and B was acetonitrile/water (90:10 v/v). Elution started with A:B (80:20%) for 0.5 min and continued with a linear gradient reaching A:B (20:80%) over 7 min. The column was then washed for 1 min with B (100%) and then returned to the initial condition. The concentration of carrier PBR28 in plasma was determined from a calibration curve (Supplementary Fig. S4), a plot of reference PBR28 concentration versus peak area ratio for reference PBR28 to the IS. The data were corrected for the skewed ratio of [^13^C]_i_ to [^12^C]_i_ in carrier PBR28 using the equation (100 + x)/(100 + y) × c, where x is ratio of [^13^C]_i_ to [^12^C]_i_ in carrier PBR28, y is the ratio of [^13^C]_i_ to [^12^C]_i_ in PBR28 (Table 1), and c is the measured concentration of carrier PBR28.

### Comparison of different types of AIF measurement for their impact on kinetic modeling

To examine the impact of different types of AIF measurement on the input function for kinetic modeling of PET data, we calculated AUCs (by trapezoidal method) from the LC–MS/MS and radiometric methods (corrected and uncorrected). The resultant AUCs data for eight subjects and their mean ± SD and relative standard deviation [RSD (%)] were tabulated for each method. Similarities between LC–MS/MS and the radiometric methods were assessed with paired samples *t* test, test–retest variability (VAR, absolute difference between methods divided by their mean value), Pearson correlation coefficients, and intraclass correlation coefficients (ICC). The data were analyzed using IBM SPSS Statistics for Mac (version 26, copyright IBM Corporation 2019). *P* values less than 0.05 were considered statistically significant.

## Supplementary information

Supplementary Information.
