# Supplementary material for: Development of a non-radiometric method for measuring the arterial input function of a 11C-labeled PET radiotracer
Source: Sci Rep. 2020 Oct 15;10:17350. doi: 10.1038/s41598-020-73646-4 (PMC7562706; doi:10.1038/s41598-020-73646-4)
Supplement: Supplementary file 1 — Supplementary Information. [file 41598_2020_73646_MOESM1_ESM.pdf]

Supplementary Information

Development of a non-radiometric method for measuring the arterial input function of a ^{11}C-labeled PET radiotracer

H. Umesha Shetty[1], Sami S. Zoghbi[1], Cheryl L. Morse[1], Aneta Kowalski[1], Jussi Hirvonen[2], Robert B. Innis[1], and Victor W. Pike[1]

[1]Molecular Imaging Branch, National Institute of Mental Health, National Institutes of Health, Bethesda, MD 20892; and [2]Department of Radiology and Turku PET Centre, University of Turku and Turku Central Hospital, FIN-20520 Turku, Finland.

Table of Contents

Supplementary Methods and Figures	Page
Materials	S2
Radiosynthesis	S2
Measurement of A_m using HPLC and an ionization chamber	S3
Technical aspects of AIF measurement by literature radiometric method	S4
Preparation of PBR28 and [^{13}C,^{2}H$_3$]PBR28 (IS) stock solutions	S4
Extraction of carrier PBR28 in plasma for LC-MS/MS analysis	S4
Recovery, matrix effect, stability, and reproducibility	S5
References	S6
Figure S1. Plot of % ratio of [^{13}C]$_i$ to [^{12}C]$_i$ in carrier versus A_m (from Table 1)	S7
Figure S2. LC-MS/MS ion chromatograms for [^{11}C]PBR28	S8
Figure S3. Scatter plots of deviation (%) in A_m values of [^{11}C]PBR28	S9
Figure S4. Calibration curve for LC-MS/MS determination of carrier PBR28	S9
Figure S5. Radiochromatograms from HPLC analysis of plasma samples	S10
Figure S6. UV/radio-HPLC analysis of [^{11}C]PBR28	S10

Supplementary Methods

Materials. Reference PBR28 (> 99% pure) was obtained from RTI International (Research Triangle Park, NC), (*R*)-rolipram from Tocris (Ellisville, MO), and (*R*)-PK11195 from ABX (Radeberg, Germany). DPA713 was synthesized in our laboratory as previously described[1]. The internal standard (IS) [^{13}C,$^{2}H_3$]PBR28 was prepared by treating *N*-(2-hydroxybenzyl)-*N*-(4-phenoxypyridin-3-yl)acetamide[2] with [^{13}C,$^{2}H_3$]methyl iodide (Cambridge Isotope Laboratories; Andover, MA). Liquid chromatography-tandem mass spectrometry (LC-MS/MS) and reagent grade solvents were obtained from Sigma-Aldrich (St. Louis, MO). Calibrated pipettes (Gilson; Middleton, WI) and aerosol-resistant tips were used for pipetting plasma and solutions of analytical standards. GraphPad Prism 8 (La Jolla, CA) software was used to plot the ratio of radioactivity to total mass (A_m) and arterial input function (AIF) data.

Radiosynthesis. The radiotracers [^{11}C]PBR28, [^{11}C](*R*)-rolipram, [^{11}C](*R*)-PK11195, and [^{11}C]DPA713 were synthesized as described in Investigational New Drug Applications 76,441, 73,149, 101,908, and 116,950, respectively. Briefly, [^{11}C]PBR28, [^{11}C](*R*)-rolipram, and [^{11}C]DPA713 were labeled with carbon-11 by treating their respective *O*-desmethyl precursors with [^{11}C]methyl iodide in the presence of tetrabutylammonium hydroxide in dimethylformamide (DMF) for 5 minutes at 22 °C. [^{11}C](*R*)-PK11195 was labeled by treating its *N*-desmethyl precursor with [^{11}C]methyl iodide in the presence of potassium hydroxide in dimethyl sulfoxide at 80 ºC. Radioligands were isolated with reversed phase high performance liquid chromatography (HPLC), evaporated to dryness on a rotary evaporator, and reconstituted for intravenous injection in sterile saline for [^{11}C]PBR28 and [^{11}C](*R*)-rolipram and in sterile saline-10% (v/v) ethanol for [^{11}C]DPA713 and [^{11}C](*R*)-PK11195. The radiochemical purities

were >99% for [^{11}C]PBR28, [^{11}C](*R*)-rolipram, and [^{11}C]DPA713, and >98% for [^{11}C](*R*)-PK11195. The radioactivity of each radiotracer was decay-corrected to the respective time of end of synthesis.

Measurement of A_m using HPLC and an ionization chamber. The ionization chamber (Atomlab 300; Biodex, Shirley, NY) in the production laboratory was calibrated using surrogate isotope reference standards of cobalt-57 and cesium-137 (Eckert & Zeigler Isotope Products, Valencia, CA). This chamber was cross-calibrated to a reference ionization chamber (CRC-15R; Capintec, Florham Park, NJ) used to measure the dose of radiotracer injected into human subjects for PET imaging. Freshly prepared and formulated [^{11}C]PBR28 solution (100 μL), contained in a syringe, was measured for radioactivity and then injected onto HPLC (Beckman, Fullerton, CA). The radiotracer was eluted isocratically at 6 mL/min with acetonitrile-aq. 10 mM HCO_2NH_4 (30: 70 v/v) on a reversed phase column (Onyx Monolithic C18; Phenomenex, Torrance, CA) equipped with radioactivity and absorbance detectors (UV 220 nm). A pair of chromatograms from the UV absorbance/radio-HPLC analysis of [^{11}C]PBR28 is shown in Fig. S6. Other radiotracers were analyzed by the same general process using suitable analytical methods. The mass of the carrier in the injectate was determined from a linear calibration curve generated from injections of known amounts of authentic non-radioactive standards. The masses of carrier detected were well above the limits of detections of the methods. Thus for [^{11}C]PBR28, calibration curves were constructed using injections in the range of 40 to 300 ng. They showed no consistent deviation from linearity at the lowest points. Historically, our radiolabeled samples have an average injected carrier amount of 95 ± 71 ng (n = 434; range 17–694 ng). The A_m was calculated from the decay-corrected activity present in the same volume of injectate, as measured in the calibrated ionization chamber.

Technical aspects of AIF measurement by literature radiometric method. The γ-counter was cross calibrated with the clinical ionization chamber. All whole blood and plasma samples were counted in polypropylene test tubes with volumes of no more than 1.0 mL. Sample volume up to 1.0 mL showed no loss of counts. Furthermore, sample volumes were selected so that the observed counts were not less than 2000 cpm and therefore had less than 3% error. The γ-counter was linear up to 18 kBq which gave a dead-time factor of 1.01. Therefore, all sample counts were accepted if the dead-time factor did not exceed 1.01. Examples of plasma radiochromatograms are provided in Fig. S5.

Preparation of PBR28 and [^{13}C,$^{2}H_3$]PBR28 (IS) stock solutions. A primary stock solution of PBR28 (500 μg/mL; DMF) was prepared and stored at 4 °C in a sealed vial (Teflon-lined cap). A secondary standard solution (10 μg/mL) was used to prepare the calibration curve for quantifying carrier PBR28 in plasma. The accuracy of the standard was verified by preparing two additional stock solutions of reference PBR28 (involving a different chemist and time) and LC-MS/MS measurement of peak areas of reference PBR28 relative to the IS. IS stock solution was similarly prepared and stored. On the day of analysis, the PBR28 solution (10 μg/mL) was appropriately diluted for preparing calibration curve samples. An IS solution (20 pg in 50 μL) was prepared and used to spike the plasma samples.

Extraction of carrier PBR28 in plasma for LC-MS/MS analysis. An aliquot of plasma (200 μL) for AIF measurement was pipetted into an Eppendorf tube containing water (200 μL), DMF (50 μL), and IS solution (50 μL), and then mixed by vortexing. The samples for constructing a calibration curve were prepared similarly by spiking the baseline plasma with solutions of reference PBR28 and IS. When prepared, these plasma samples typically contained 400–1.5625

pg/mL (1148.9−4.488 fmol/mL) of PBR28. Both sets of plasma samples were stored at − 75 °C and analyzed within one week. On the day of analysis, the thawed plasma samples were transferred to glass tubes (with Teflon-lined cap) by pipetting. The residual plasma in the Eppendorf tube was recovered by rinsing it with water (200 µL) and transferring the rinsate to the same glass tube. Sodium carbonate solution (10% w/v; 100 µL) and ethyl acetate−*n*-hexane mixture (90:10; v/v; 2.5 mL) were added to each tube, mixed vigorously (15 s) and then centrifuged (1000*g*; 1 min). The aqueous layer was frozen (dry-ice bath) and the mobile organic layer was decanted immediately to a glass tube. To prevent cross-contamination, the mouth of the tube was covered with filter paper (Whatman no. 1; ~ 2 in diameter) and sealed with Scotch tape. The solvent in the tube was evaporated off at 22 °C with a concentrator (SpeedVac; Thermo Fisher Scientific, Waltham, MA), and the residue reconstituted in aq. acetonitrile (50% v/v) containing 1% acetic acid (200 µL). The extract was filtered (4 mm × 0.2 µm; PTFE-4-2; Sigma-Aldrich) into an autosampler vial, and a sample (10 µL) was then injected onto LC-MS/MS for analysis, as described previously (Methods section of the paper).

Recovery, matrix effect, stability, and reproducibility. *Recovery:* A set of plasma samples (200 µL; *n*=5) containing PBR28 (10 pg) was prepared and extracted with ethyl acetate−*n*-hexane mixture. After freezing the aqueous layer, the organic layer was transferred to another glass tube containing the IS solution. The plasma extract was processed and PBR28 quantified with LC-MS/MS. The liquid-liquid extraction recovered 93.2±2.3% of PBR28 from the plasma. *Matrix effect:* The effect of plasma matrix on MS/MS ionization and detection was evaluated by analyzing PBR28 plus IS prepared in plasma extract and in solvent only as a control (*n*=3 each). The ratio of PBR28 to IS peak area was unaltered in samples prepared in plasma extract.

Stability: Stability of carrier PBR28 in plasma was assessed from a PET experiment with [^{11}C]PBR28. Plasma from each of the 12 timepoints was divided to generate a batch of samples with the added IS (Set 1) and another without it (Set 2). After one week of storage (− 75 °C), thawed samples from Set 2 were mixed with IS. Subsequently, carrier PBR28 was quantified in Set 1 and 2 samples. The concentrations of carrier PBR28 in each of the Set 2 plasma samples differed between −0.12 and +5.23% (mean: ±1.81%) from the corresponding Set 1 samples, and the difference between the two sets was insignificant (P>0.05).

Reproducibility: Plasma samples (200 µL; n=3) from each of two timepoints of a PET experiment with [^{11}C]PBR28 were spiked with the IS, stored, and then analyzed with LC-MS/MS. The concentrations of carrier PBR28 in these samples were 63.83±0.36 (RSD=0.56%) and 15.47±0.22 (RSD=1.42%) pM, respectively. In addition, plasma (n=4) containing 287.2 and 8.98 pM reference PBR28 were prepared and analyzed. These two sets of samples showed 288.3±6.1 (RSD=2.1%) and 8.73±0.43 (RSD=5.0%) pM of reference PBR28, respectively.

References

1. Selleri, S. *et al*. 2-Arylpyrazolo[1,5-*a*]pyrimidin-3-yl acetamides. New potent and selective peripheral benzodiazepine receptor ligands. *Bioorg. Med. Chem.* **9**, 2661–2671 (2001).
2. Briard, E. *et al*. Synthesis and evaluation in monkey of two sensitive ^{11}C-labeled aryloxyanilide ligands for imaging brain peripheral benzodiazepine receptors in vivo. *J. Med. Chem.* **51**, 17−30 (2008).

Supplementary Figures

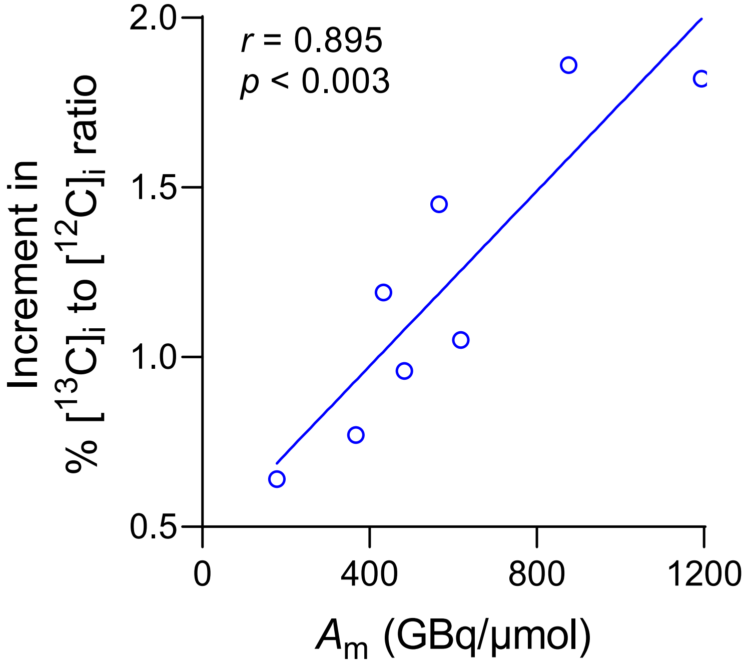

Figure S1. Increment in percentage ratio of $[^{13}C]_i$ to $[^{12}C]_i$ in carrier versus A_m (measured radiometrically; decay-corrected to the time of end of synthesis) for four different radiotracers (from Table 1 data).

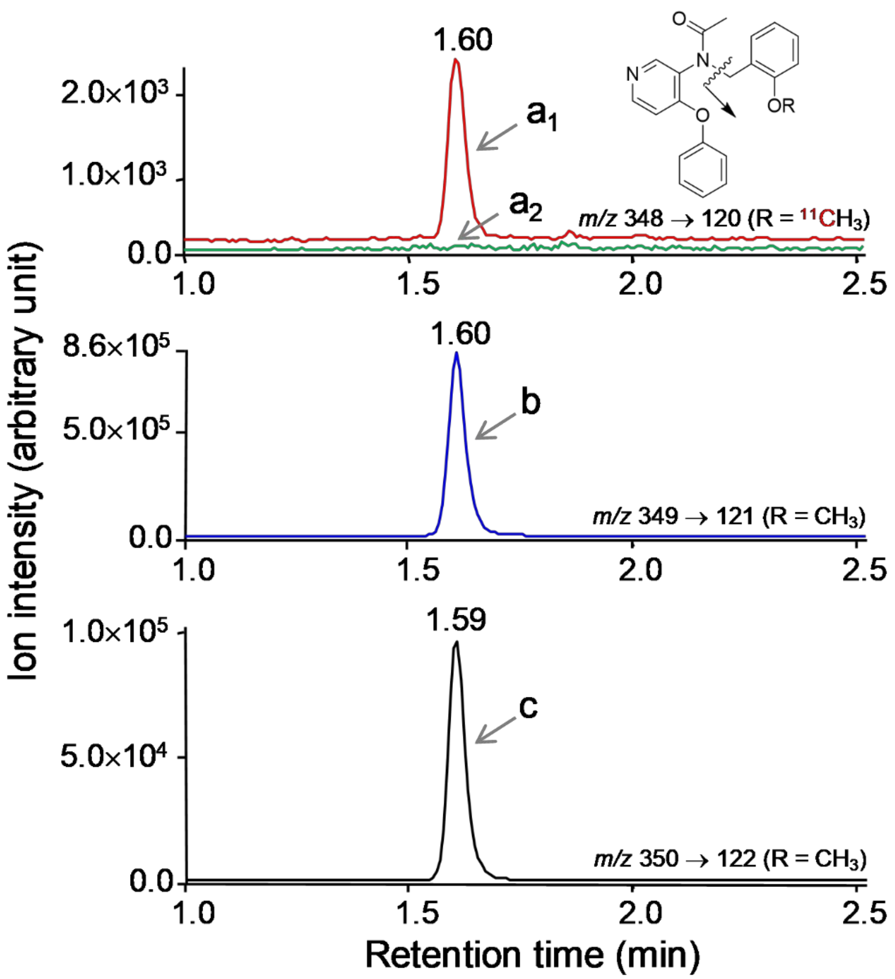

Figure S2. Examples of LC-MS/MS ion chromatograms for [^{11}C]PBR28 with a high A_m value (1124 GBq/µmol). Top: [^{11}C]$_i$ at baseline (**a₁**) and after its full decay (**a₂**); middle:[^{12}C]$_i$ (peak **b**); and bottom [^{13}C]$_i$ (peak **c**).

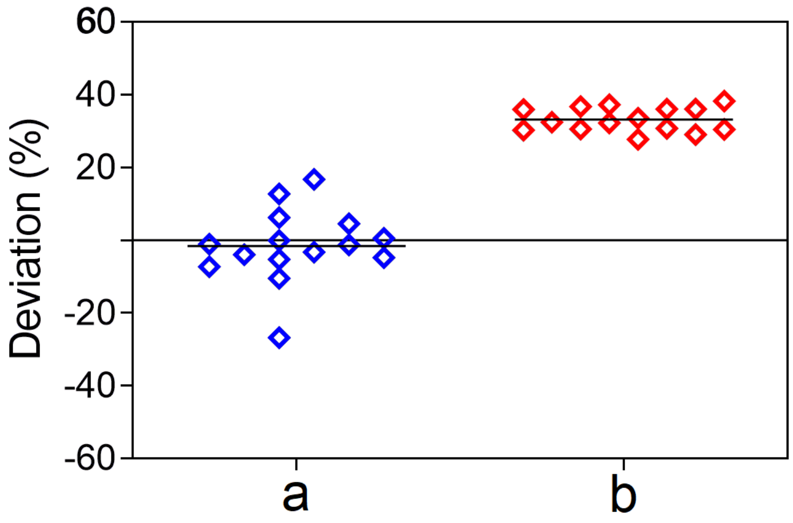

Figure S3. Scatter plots of deviation (%) in A_{m} values of [^{11}C]PBR28 measured (**a**) with an ionization chamber (Method 2), and (**b**) with a γ-counter (Method 3) from the values obtained from LC-MS/MS alone (Method 1).

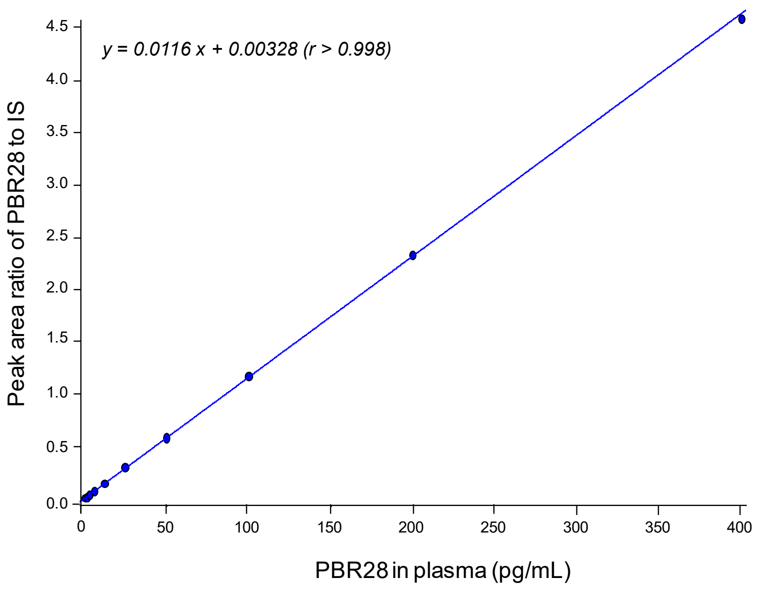

Figure S4. Calibration curve for LC-MS/MS determination of carrier PBR28 in the plasma of human subjects injected intravenously with [^{11}C]PBR28.

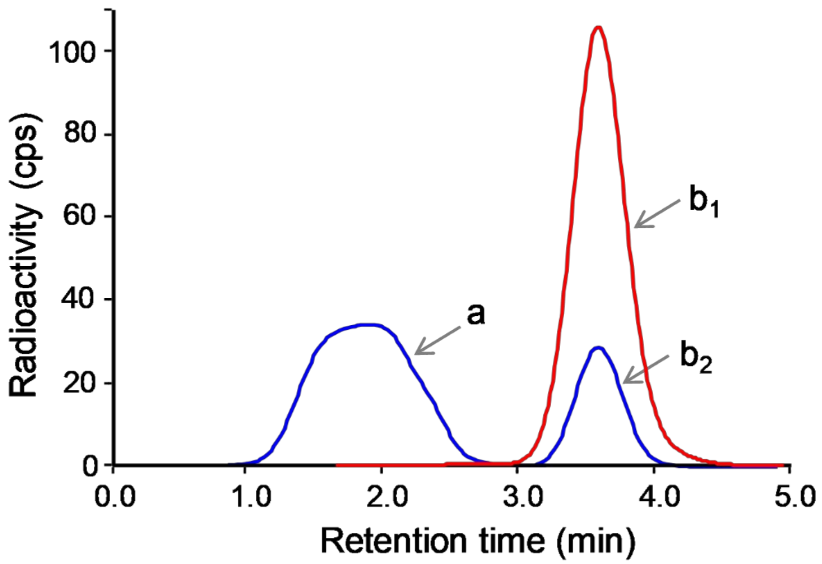

Figure S5. Radiochromatograms from HPLC analysis of plasma samples from a human subject injected with [^{11}C]PBR28 (138.6 GBq/µmol). Peak $\mathbf{b_1}$, parent radiotracer only from plasma sampled 0.5 min after radiotracer injection into subject. Peak $\mathbf{b_2}$, radiotracer, and peak **a**, radiometabolites from plasma sampled at 50 min after radiotracer injection into subject.

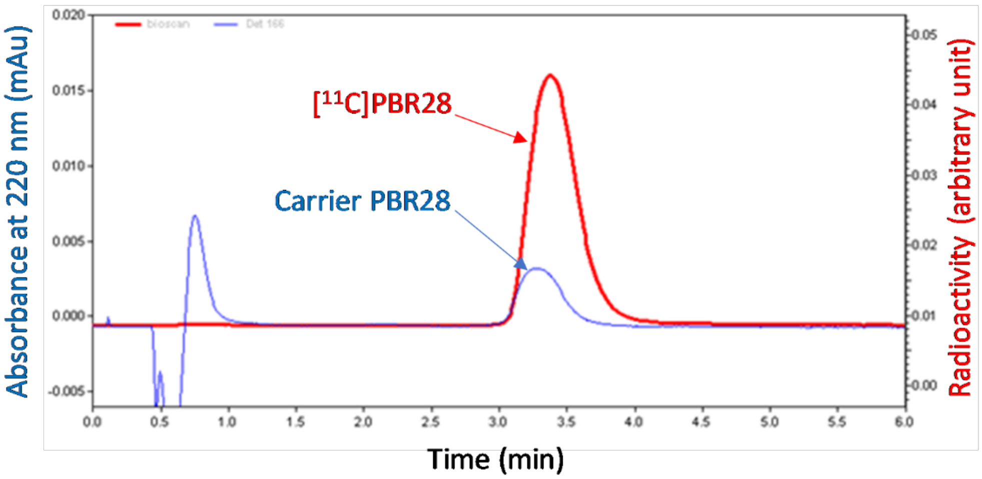

Figure S6. UV/radio-HPLC analysis of [^{11}C]PBR28: dual chromatograms showing detection of [^{11}C]PBR28 (radiochemical) and its carrier (UV).
